# Autologous tumor lysate/*Bacillus Calmette*–*Guérin* immunotherapy as an adjuvant to conventional breast cancer therapy

**DOI:** 10.1007/s12094-015-1320-0

**Published:** 2015-06-16

**Authors:** J. Convit, H. Montesinos, H. Oviedo, G. Romero, B. Maccarone, E. Essenfeld, A. Convit, L. E. Palacios

**Affiliations:** Instituto de Biomedicina Dr. Jacinto Convit, Distrito Capital, Venezuela; Universidad Simón Bolívar, Sartenejas, Distrito Capital, Venezuela; Hospital Oncológico Dr. Luis Razetti, Distrito Capital, Venezuela; Cirugía General & Mastología, Policlínica Metropolitana, Distrito Capital, Venezuela; Anatomía Patológica, Policlínica Metropolitana, Distrito Capital, Venezuela; Fundación Jacinto Convit, Caracas, 1071 Venezuela

**Keywords:** Immunotherapy, Autologous, BCG, Vaccine, Breast cancer

## Abstract

**Introduction:**

Autologous tumor cell vaccines rely on the concept of preserving an individual’s own tumorigenic makeup, expressing its unique set of tumor-associated antigens as well as antigenic elements from the surrounding stroma. These autologous tumor characteristics are usually presented with an immune adjuvant in the hopes of enhancing an immune response.

**Methods:**

The autologous vaccine we used was composed of tumor cells combined with BCG and formalin. Animal safety and toxicity were evaluated using mice tumors for the immunotherapy. A small number of patients with advanced stage breast cancer were recruited for an uncontrolled study, using the vaccine solely or combined with chemotherapy/radiotherapy.

**Results:**

The immunotherapy had shown to be safe in mice and humans. Upon a 5-year follow-up, the survival rate was 60 % for the combined treatment.

**Conclusions:**

The data suggest that the combined treatment could be a feasible and safe therapeutic strategy. However, further controlled studies should be conducted.

## Introduction

Breast cancer is one of the most frequent cancers of women worldwide, and among the major causes of death. The rate of breast cancer local relapse is 5–45 % after surgery [1, 2]. Therefore, post-operative radiotherapy or other systemic treatment is utilized to prevent metastases. There is a need for adjuvant treatments for tumor and metastasis control [3].

Improved immune system status might be important in the prevention of relapse, since certain depression of cell-mediated immunity may occur during the development of breast cancer [4]. Chemotherapy and radiotherapy may further contribute to immunological depression. Therefore, immunotherapeutic approaches using potential adjuvant treatments to prevent breast cancer relapse have been considered.

Molecular immunology advances have led to the discovery of multiple tumor antigens and pathways, as well as many other immunoregulatory aspects of breast cancer [5]. Molecular antigenic profiling of neoplastic breast cells have revealed variable patterns of tumor antigen expression, posing a challenge on the utilization of single antigenic targets for the development of specific immunotherapies [6, 7]. Conversely, whole cell-based vaccines, despite their higher, but rare, risk of developing autoimmune phenomena, may provide a broader load of antigenic components capable of inducing a broader and more effective immune response [8–10].

The use of BCG in immunotherapy of various tumors has been well studied [11]. It is known that BCG phagocytosis induces liberation of pro-inflammatory cytokines with the subsequent development of a Th1 reaction [12, 13]. Similar responses have been revealed in chronic infectious diseases, such as lepromatous leprosy and Leishmaniasis. We had found that BCG injected with a *Mycobacterium leprae* suspension induced macrophage activation and subsequent destruction of *M. leprae* [14, 15]. Additionally, an immunotherapy to treat cutaneous leishmaniasis was designed, using pasteurized *Leishmania* promastigotes with BCG [16]. Both therapies have been used with positive results. When coupled with the minimal side effects associated with BCG, the efficacy shown in these studies supports the use of BCG for other applications.

Based on these positive experiences and assuming that the relapse of breast cancer could be partially due to a lack of appropriate recognition of tumor cells as foreign, the use of own tumor cells with BCG as an adjuvant immunotherapy for breast cancer was previously proposed by Convit and Ulrich [17]. Here, we present the results of an uncontrolled study of a small number of patients in advanced stage of the disease, using the individuals’ own tumor tissue to produce a personalized vaccine. Furthermore, we report the proven safety of this treatment, as well as a promising positive result for the use of this immunotherapy.

## Materials and methods

### Animal safety study

Fifteen female six-week-old BALB/c mice were divided into three groups of five (5) animals. Group 1 included animals treated tumor-free, group 2 untreated tumor-induced, and group 3 treated tumor-induced. For vaccine preparation, tumors extracted from previously induced mice were used. Tumors were induced injecting intradermally 8 × 10^5^ 4T1 cells per mouse into the mammary gland. Three doses of the treatment were applied intradermally on their back every week, starting 15 days after induction when tumors were palpable. Examination of the general condition of the animals and local reaction in the area of vaccination was performed every 2 days for 1 month, then twice a week for the next 2 months. Additionally, female BALB/c mice, hamsters and guinea pigs were immunized intradermally on their back using a homogenate from human mammary tumor plus BCG and formalin. Their general condition and local reaction were evaluated.

### Human study

For the small uncontrolled study, twenty (20) patients in advanced stages with limited life expectancy were recruited in a 2-year period. Patients were classified into two groups according to the treatment to be employed. Group 1 included 6 patients who refused to be treated with standard treatments and received only immunotherapy. Group 2 included 14 patients treated with both immunotherapy and chemotherapy/radiotherapy (CT/RT). All patients referred to this study gave their informed consent, and were aware that their unidentified data would be used for publication. This study was duly approved and cleared by the Ethics Committee of “Dr. Luis Razetti” University Hospital of Barcelona, Venezuela.

The vaccination procedure included three intradermal doses of 0.5 ml each, with intervals of 6 weeks between each dose. Tumor fragments (about 1 g) from patients were kept in sterile PBS (4.45 mM Na_2_HPO_4_, 1.55 mM NaH_2_PO_4_, 137 mM NaCl, pH 7.2) at −80 °C until use. For each dose, about 0.3–0.4 g of tumor tissue were macerated in 0.6 ml sterile PBS. At the moment of vaccination, the soluble fraction of the homogenate was mixed with formaldehyde 36 % at a final concentration of 0.02 % and 0.6 ml of BCG suspension 0.15 % (Staten Serum Institute, Copenhagen).

## Results

### Animal studies

All treated tumor-free animals (Group 1) were still alive within 3 months after induction, while all untreated tumor-induced animals (Group 2) developed palpable tumors and died within 45 days after induction. Only two (2) of the treated tumor-induced animals (Group 3) were still alive 3 months after induction with intangible tumors, representing a 40 % survival. Both treated groups showed a normal growth rate and no local reaction was apparent. Same results were observed on those mice, hamsters and guinea pigs that were immunized with the human vaccine. These observations led us to consider that the immunotherapy was tolerable for the animals, and that it was safe and non-toxic.

### Human study

**Fig. 1:**
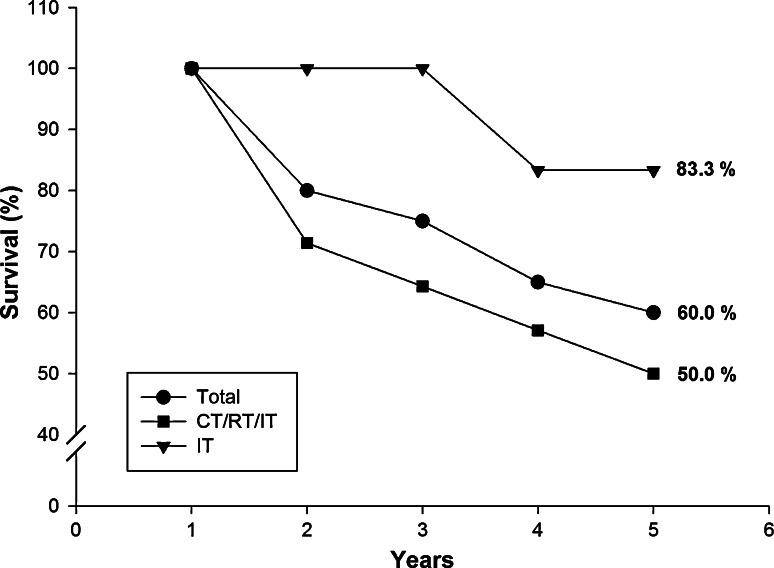
Estimated survival rates. The survival rate upon a 5-year period was plotted for the immunotherapy only (IT) group (*inverted filled triangle*) and the combined treated (CT/RT/IT) group (*filled square*), as well as for the overall survival across all subjects (*filled circle*). The calculated 5-year survival is indicated for each plot

Seeking to contrast standard treatment commonly used in patients with the immunotherapy proposed here, we calculated the estimated overall survival. For the immunotherapy-only group, the estimated survival was 83.3 %, while for the combined treatment (CT/RT/IT) was 50 %, resulting in an overall survival of 60 % across all subjects (Fig. 1). The high estimated survival obtained for the immunotherapy-only group might be due to the small number of subjects, as compared with the combined treated group. The calculated overall survival rate (60 %) and that for the combined treatment (50 %) are similar to the estimated survival reported by Parkin et al. [18] of 67 % for females with breast cancer in Latin America and the Caribbean. These results indicate that the immunotherapy proposed (solely or combined) was similarly effective to the standard treatment, and was considered safe and non-toxic. To reach a more significant conclusion on its effectiveness, a more specific analysis of the immunological effect involving a higher number of patients is necessary.

## Discussion

For cancer solid tumors, surgery has become the potentially curative treatment choice available; while for patients with advanced stages or metastatic disease, this is rarely sufficient [19]. Conventional CT or RT provides acceptable response rates and improved survival, but they generally lack tumor specificity and induce cytotoxicity in normal cells. Alternative therapeutic strategies with fewer side effects are being studied. The immune system is characterized by high specificity, and takes part in the formation of a tumor’s microenvironment, contributing to tumor elimination and progression. In recent years, immunotherapy has been finely explored as a new form of treatment [19, 20].

The low antigenicity and depleted capacity of tumor cells in stimulating antigen presentation are well known, as well as the inter- and intra-tumoral heterogeneity. Therefore, vaccination models are usually based on whole autologous or allogeneic tumor cells; both being used in clinical trials for breast cancer treatment with variable reports of improved survival [8, 21–23]. As far as adjuvants in cancer vaccines, microbes have been utilized for this purpose as they may break immunological tolerance toward self-antigens. The use and benefits of BCG as an adjuvant in the immunotherapy for various types of tumors have been widely supported [19, 24], and this justified its use here.

As part of the immunologic approach, we made an indirect evaluation of the immune system response in all patients before and after treatment using the intradermal tuberculin test (PPD for purified protein derivative). We identified a boost of the immune response in all patients after treatment, despite the concomitant application of CT and/or RT (data not shown). Whether this augmentation of cell-mediated immunity is directly related to the survival rates observed will need to be established in a larger study.

Although the low number of patients used in this study might not be statistically significant to make accurate conclusions, the calculated overall survival rate is similar to that previously reported for standard treatment [18]. Please note that this equivalency with the overall statistics was present despite all patients in this uncontrolled trial having advanced metastatic disease. Therefore, one could conclude that the combined treatment (IT/CT/RT) is at least similarly effective as the conventional treatment alone, but with lower occurrence of side effects, allowing patients to have a better quality of life. Since our proposed immunotherapy uses a simple low-cost method of preparation, it could be a feasible and safe immuno-therapeutic resource, which coupled with surgery and/or systemic CT/RT may reduce the recurrence of the disease. This work may contribute to the recent worldwide efforts to study immunotherapy as a new alternative treatment for cancer, but further studies should be performed to reach firmer conclusions.

## Abbreviations

*BCG*: *Bacillus Calmette*–*Guérin*
IT: Immunotherapy
CT: Chemotherapy
RT: Radiotherapy
